# Antigenic variation in vector-borne pathogens.

**DOI:** 10.3201/eid0605.000502

**Published:** 2000

**Authors:** A G Barbour, B I Restrepo

**Affiliations:** University of California Irvine, Irvine, California 92697-4025, USA. abarbour@uci.edu

## Abstract

Several pathogens of humans and domestic animals depend on hematophagous arthropods to transmit them from one vertebrate reservoir host to another and maintain them in an environment. These pathogens use antigenic variation to prolong their circulation in the blood and thus increase the likelihood of transmission. By convergent evolution, bacterial and protozoal vector-borne pathogens have acquired similar genetic mechanisms for successful antigenic variation. Borrelia spp. and Anaplasma marginale (among bacteria) and African trypanosomes, Plasmodium falciparum, and Babesia bovis (among parasites) are examples of pathogens using these mechanisms. Antigenic variation poses a challenge in the development of vaccines against vector-borne pathogens.


### Antigenic Variation in Vector-Borne Pathogens

**Alan G. Barbour* and Blanca I. Restrepo†**
*University of California Irvine, Irvine, California; and
†Corporación para Investigaciones Biológicas, Medellín, Colombia

Several pathogens of humans and domestic animals depend on hematophagous arthropods to transmit them from one vertebrate reservoir host to another and maintain them in an environment. These pathogens use antigenic variation to prolong their circulation in the blood and thus increase the likelihood of transmission. By convergent evolution, bacterial and protozoal vector-borne pathogens have acquired similar genetic mechanisms for successful antigenic variation. *Borrelia* spp. and *Anaplasma marginale* (among bacteria) and African trypanosomes, *Plasmodium falciparum*, and *Babesia bovis* (among parasites) are examples of pathogens using these mechanisms. Antigenic variation poses a challenge in the development of vaccines against vector-borne pathogens.

### What Is Antigenic Variation?

Immunodominant antigens are commonly used to distinguish strains of a species of pathogens. These antigens can vary from strain to strain to the extent that the strain-specific immune responses of vertebrate reservoirs determine the population structure of the pathogen. One such strain-defining antigen is the OspC outer membrane protein of the Lyme disease spirochete *Borrelia burgdorferi* in the northeastern United States (1). Different strains express different OspC surface proteins in the rodent reservoirs of *B. burgdorferi*. The single type of *ospC* gene in a cell does not vary during infections of immunocompetent mammals (2). OspC sequences are diverse, and the immune responses to them appear to provide for balancing selection. This diversity between strains in an immunodominant antigen is often called antigenic variation.

True antigenic variation, however, arises in a single clone or genotype in a single host and "involves the loss, gain, or change in a particular antigenic group, usually by loss, gain, or change in one of the polypeptide or polysaccharide antigens..." (3). In most cases, this change is reversible, i.e., the information for producing the original antigen is archived in the cell and can be used in the future. The adaptive immune system of an infected vertebrate selects against the original infecting serotype, but that specific response is ineffective against new variants. One example of antigenic variation occurs in *B. hermsii*, a cause of tickborne relapsing fever (4), which has a protein homologous to the OspC protein of *B. burgdorferi*. However, instead of a single version of this gene, each cell of *B. hermsii* has several copies of silent genes (alleles) that may be expressed during infection. The sequences of these alleles within a single strain of *B. hermsii* vary as widely as the *ospC* alleles of different strains of *B. burgdorferi*.

We review infectious pathogens that undergo clonal antigenic variation and, like *B. hermsii*, depend on arthropod vectors for transmission. These pathogens are not free-living and do not form spores or have equivalent means for survival outside an animal. Vertical transmission in the arthropod or the vertebrate either does not occur or is too rare to maintain the pathogen in nature. Without access to another vertebrate host through an arthropod, the pathogen will die with the host.

We restrict this review to situations in which an immune response against an antigen is synonymous with selection for another allele in the population. Many pathogens have repetitive-gene families. A multimember family may

Address for correspondence: Alan Barbour, Department of Microbiology & Molecular Genetics, University of California Irvine, Irvine, CA 92697-4025; fax: 949-824-5626; e-mail: abarbour@uci.edu.

resemble a variable antigen gene repertoire in its diversity but may have little or no effect on immunity against infection. An example is the *bdr* family of genes in *B. burgdorferi* (5).

Viruses are also said to have antigenic variation but are excluded from this review because the mechanism they use usually depends either on the accumulation of point mutations in a single genotype (e.g., the antigenic drift of influenza A virus) or on recombination or reassortment between two different genotypes infecting the same host (e.g., antigenic shift of influenza A virus). A possible exception is the African swine fever virus, a poxvirus-like linear DNA virus transmitted by soft ticks. These large viruses have tandem repeated genes at their telomeres that undergo deletions during infection (6).

### Common elements

Infection with a vector-borne pathogen that undergoes clonal antigenic variation has several possible outcomes (Figure 1). Acquisition of the pathogen by the vector does not in itself constitute transmission. The vector may become infected by a blood meal, but enough pathogens may not be present in the blood for the vector to transmit the infection to its next host. Pathogen peaks may not be so well delineated, especially during late infection when the growth-and-decline curves for individual variants begin to overlap.

*Borrelia* spp., *Anaplasma* and related genera, African trypanosomes, *Plasmodium* spp., and *Babesia* spp. are vector-borne pathogens that use antigenic variation to evade the host's immunity. The details of antigenic variation differ, but some features are the same, for example, the use of multiphasic antigenic variation or a change among at least three variable antigens rather than alternating between two. At least 10—and sometimes many more—variants or serotypes may be expressed during a single infection. There is a complete or near-complete gene for each of the variable proteins. Variation is achieved by switching one of the several genes expressed at any one time, rather than by accumulating mutations in a single expressed gene, as commonly occurs in viruses. In the best-studied examples, African trypanosomes and relapsing fever *Borrelia* spp., the rate of antigen switching in the vertebrate host is approximately the same, at $10^{-4}$ to $10^{-2}$ per cell per generation.

True antigenic variation has been demonstrated in other human pathogens, including *Neisseria gonorrhoeae*, *Mycoplasma* spp., *Campylobacter fetus*, *Pneumocystis carinii*, and *Giardia lamblia*. In addition, the complete genomic sequences of other pathogenic bacteria, such as *Helicobacter pylori*, *Treponema pallidum*, and

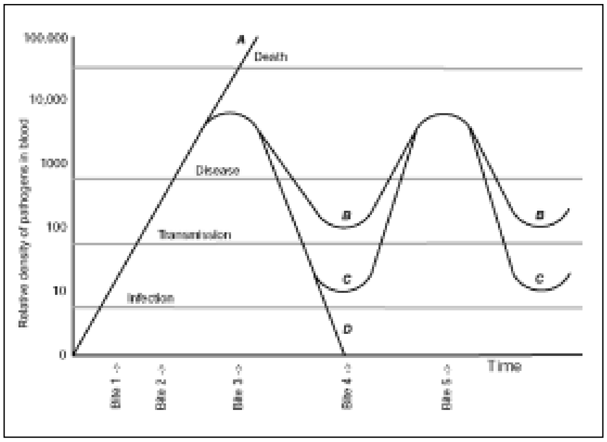

Figure 1. Relative densities of a vector-borne pathogen in the blood of four hosts, A-D. The gray horizontal lines represent the lower thresholds for the persistence of infection, transmission to a new vector, symptomatic disease in the host, and death of the host. In host A, overwhelming infection causes death. In hosts B and C, there is antigenic variation of the pathogen; B differs from C in the continuing likelihood of transmission even during periods of no or little illness. In case C, the host remains infected but is not infectious between relapses of high-density parasitemia and illness. In host D, the pathogen is cleared from the blood by the immune system. The relative density of the pathogens in the blood is on the *y* axis. If the arthropod vector of the infection fed on the four hosts at different times, the following outcomes would be observed: a bite at time 1 would not result in transmission because the density of pathogens in the blood was insufficient for uptake and establishment of the pathogen in the vector. At times 2 or 3, the disease agent is transmitted to the biting arthropod, although the hosts bitten at time 2 are not yet ill. At time 3, the infection worsens, in case A in the absence of an effective immune response. In cases B-D, the infection peaks as neutralizing antibodies to the infecting serotype appear in the blood at time 3. An arthropod taking a blood meal at time 4 could acquire the infection from host B but not from hosts C or D. In case D, the infection has been cleared by time 4. At time 5, both B and C could transmit the pathogens again and both have a relapse of illness as the result of the proliferation of a new serotype. The figure is modified from a figure by Turner (7).

*Mycobacterium tuberculosis*, encompass large families of repeated genes that are polymorphic in sequence and may be involved in antigenic variation.

The vector-borne pathogens, in particular the African trypanosomes and relapsing fever *Borrelia* spp., offer the least ambiguous models for understanding the biology and evolution of antigenic variation. These pathogens depend on hematophagous arthropods for transmission to new vertebrate hosts, and consequently, the likelihood of transmission is a direct function of the duration and density of the pathogens in the blood. Blood-cell types are comparatively simple, and antibodies alone can clear infection by relapsing fever *Borrelia* spp. and African trypanosomes (8,9). Apparently these two pathogens need to defend only against humoral immunity.

Vector-borne pathogens use one or more genetic mechanisms to circumvent the immune system. Four general mechanisms for antigenic variation have been described (10): modification of transcript levels, gene conversion, DNA rearrangement, and multiple point mutations (Figure 2). An example of the first mechanism is the reversible activation of expression of a variable antigen gene at one locus as expression of a previously active variable antigen gene at another locus in the genome becomes silent, an event that occurs without DNA changes at the loci themselves. In the figure, pathogen X has surface antigen genes *black* and *white* at two loci. At each locus there is a potential promoter, but only at the *black* locus is a gene expressed. After a switch, the *black* locus is silent, but the *white* locus 3 is active.

The second mechanism, gene conversion, is probably the most widespread for replacing expression of one gene with another. The change may be complete, thereby altering all defining epitopes of the antigen, or partial, for example, when a central hypervariable region of a protein is replaced through crossovers in highly similar flanking regions. This process commonly involves genes on separate chromosomes or plasmids in the cell but can also occur within the same replicon. When one gene is displaced at an expression site, the organism uses for that replacement a copy of a gene from a more stable location in the genome. In Figure 2, a *black* gene converts a *white* gene at a site with an active promoter. Gene conversion allows a pathogen to retain a complete repertoire of variable antigen genes.

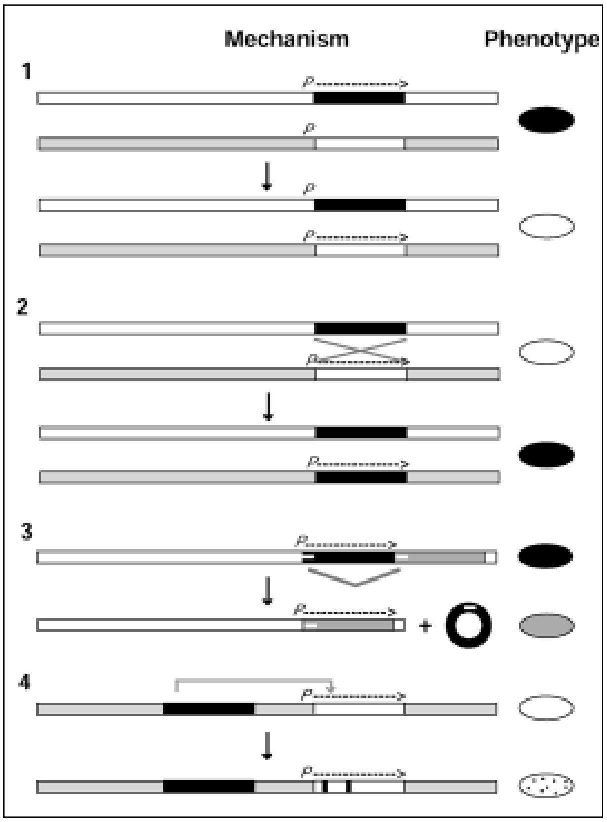

Figure 2. Four genetic mechanisms for antigenic variation in a hypothetical pathogen. 1. Modification of transcript levels. Two loci are shown in the figure for *black* and *white* genes. When the *black* gene promoter (*P*) is silenced and the *white* gene promoter is activated (arrow), the phenotype of the pathogen changes from black to white. No genetic change occurs at either locus. 2. Gene conversion. Two loci are shown for *black* and *white* genes: the *white* gene is at the expression site with a promoter, and the cell phenotype is white. In the switch, the *black* gene sequence is the donor that converts the expression site locus, and as a consequence the phenotype changes to black. 3. DNA rearrangement. In one locus with a tandem pair of variable antigen genes, *black* and *gray*, a recombination between direct repeats (small white boxes) at the 5' ends of the genes results in deletion of the *black* gene as a nonreplicative circle and the rearrangement of *gray* gene to a position immediately downstream of the promoter. The cell phenotype changes from black to gray. 4. Multiple point mutations. A single region of the genome contains an active *white* gene and an archived *black* gene at some distance 5' to it. The *black* gene provides donor sequences for two short conversion patches in the *white* gene. The phenotype of the cell remains white, but there may be several amino acid differences between this mutated *white* gene and the original *white* gene.

More extensive change in the genotype may occur by the third mechanism, DNA rearrangement. In Figure 2 the first gene in a tandem array of two variable antigen genes is deleted, thus moving a previously silent *gray* gene next to a promoter. The recombination is between two short direct repeats common to both the *black* and *gray* genes. Although this results in the loss of that particular allele of the *black* gene as a nonreplicative circle, there usually would be another copy of the *gray* allele in the genome.

In the pathogens discussed here, the fourth general mechanism, multiple point mutations or conversion patches, usually occurs in a gene that has already been activated or moved by one of the other three mechanisms. In Figure 2, the expressed *white* gene undergoes limited gene conversion by the *black* gene, in a process similar to somatic mutations of rearranged immunoglobulin genes.

In any given strain, the repertoire may have considerable sequence diversity, but this does not mean that each strain has achieved a unique solution to the problem of immune avoidance. Other strains and other species in the same genus usually have a repertoire of genes homologous to the set of genes of the pathogen in question (11). The evolutionary distance between two variable antigen genes in the same pathogens may be greater than the distance between two genes in a different species. An example would be the hypothetical variable gene repertoire A, B, C, and D in species 1 and variable gene repertoire A', B', C', and D' in species 2. The sequence identity between A, B, C, and D in species may be no more than 40%, but there may be 80%-90% sequence identity between the B gene in species 1 and the B' gene in species 2.

### Relapsing Fever *Borrelia* spp.

The antigenic variation of relapsing fever spirochetes once attracted the attention of the early immunologists, such as Paul Ehrlich, because the infection proved the specificity of the immune response (8,9). In *B. hermsii*, a New World relapsing fever species, approximately 30 serotypes have been derived from a single cell (12). Specific antisera to these serotypes accounted for approximately 80%-90% of the variants that appeared during relapses of infection in mice in prospective experiments (13). The serotype of a *Borrelia* cell depends on its major surface antigen. The 30 or so antigens are divided approximately equally between two families: Variable Large Proteins (Vlp) of approximately 36 kDa and Variable Small Proteins (Vsp) of approximately 20 kDa (14-16). These abundant lipoproteins are anchored in the outer membrane by their lipid moieties. Although the *vlp* and *vsp* genes use the same locus for expression and may have identical signal peptides, no sequence homology can be identified between these two groups of proteins. Information about the variable antigens of other relapsing fever *Borrelia* spp. is less extensive, but *B. recurrentis*, the cause of louse-borne relapsing fever (17), *B. crocidurae* (18), an Old World relapsing fever species, and *B. turicatae* (19), another New World species, have *vlp* and *vsp* genes themselves. The Vsp proteins not only serve as variable antigens that may attract attention as an immune target but also determine tissue tropisms. In a clonal population of *B. turicatae*, expression of one *vsp* gene is associated with invasion of the central nervous system, while expression of another *vsp* gene is associated with high densities of spirochetes in the blood (20, 21).

*B. hermsii* uses all four general mechanisms for antigenic variation. Gene conversion between a linear plasmid containing a collection of silent *vsp* and *vlp* genes and another linear plasmid with an active *vsp* or *vlp* gene results in the replacement of one variable antigen gene with another downstream from a promoter (22,23). The gene conversion may be less extensive, yielding a chimeric *vlp* gene from a partial gene conversion (24). In *B. turicatae*, conversion may be more extensive, involving 10 or more kilobases downstream of the promoter (19). A DNA rearrangement in which the first member of a tandem pair on a single linear plasmid is deleted also yields a new serotype in the population (25). After the deletion, the formerly distal *vsp* in the pair is now next to the promoter. This type of rearrangement may be followed by a period of hypermutation at the 5' end of the gene (26). These frequent point mutations in the newly expressed gene further diversification of the *vsp* sequences.

These three types of events occur at a single expression site in the genome. Evidence also indicates that variation may also occur through the fourth mechanism: a change in transcription between two separate loci (27). A second locus for *vsp* expression has been found in *B. hersmii* on another linear plasmid (27). When this locus is active, the first expression site is silent (28).

Activation of this second site is associated with infections of ticks, not mammals (29).

*B. burgdorferi* and related species that cause Lyme disease have, as stated above, only one copy of the *vsp* ortholog, *ospC*, per cell, but it has several copies of sequences called *vlsE* genes, which are homologous to the Vlp proteins of relapsing fever *Borrelia* spp. (30). During infection, but not detectably in vitro, there is variation in expressed VlsE proteins through partial gene conversions from a tandem array of cassettes containing different hypervariable regions of *vlsE* sequences. Variants appeared in both immunodeficient as well as immunocompetent mice, but the rate of accumulation of amino acid changes in VlsE was higher in immunocompetent animals (31).

### *Anaplasma marginale* and Related Bacteria

Anaplasmosis, a persistent intraerythrocytic infection of cattle and goats, has a global distribution. Infected animals have severe anemia and a higher rate of abortion. The infection, which is caused by members of the genus *Anaplasma*, an obligate intracellular rickettsia-like bacterium related to the genus *Ehrlichia*, is characterized by repetitive cycles of rickettsemia at 6- to 8-week intervals. In cattle infected with *Anaplasma marginale*, the number of pathogens in the blood varies between a peak of $10^6$ to $10^7$ per mL to a low of $10^2$ per mL. In each cycle, the number of pathogens in the blood increases over 10 to 14 days and then precipitously declines (32). The cattle are reservoirs for the infection, and ixodid ticks are the vectors. Transmission to the vector ticks depends on the density of the pathogens in the blood (33).

Major surface protein 2 of *A. marginale* is an immunodominant outer membrane protein of approximately 40 kDa. Each strain has a large polymorphic family of *msp2* genes; the variation occurs in the central region of the proteins. Multigene families of MSP2 paralogs have been found in *Cowdria ruminatium* (34), the cause of heartwater disease of ruminants in Africa and Caribbean, and *Ehrlichia granulocytophila*, the agent of human granulocytic ehrlichiosis (35). Each sequential cycle of rickettsemia is associated with a different transcript from at least 17 different *msp2* genes in the family (36). Vaccinating animals with recombinant MSP2 produces antibodies specific for that MSP2. The genetic mechanisms for switches in *msp2* genes are not known but may involve partial gene conversions through homologous recombination.

### African Trypanosomes

*Trypanosoma brucei* is a flagellated protozoon transmitted by tsetse flies to mammalian hosts, including humans and livestock. The infection consists of rising and falling parasitemia resulting from the generation of subpopulations that have antigenically different forms of a major variant-specific glycoprotein (VSG) at the cell surface (37). African trypanosomes switch at rates that are as low as $10^{-7}$ to $10^{-6}$ for syringe-passaged lines (38) or as high as $10^{-3}$ to $10^{-2}$ for field or fly-transmitted lines (39). VSG proteins, which are 400 to 500 amino acids in length, are anchored to the parasite's membrane at their carboxy terminus by a glycosyl-phospatidyl-inositol linkage. Besides immune evasion, other possible functions of VSGs include shielding other proteins (e.g., permeases) on the surface from immune attack and inhibiting phagocytosis (37).

A parasite can express several VSGs during infection in the mammalian host. Active genes for VSGs are located in one of 20 possible telomeric expression sites on the chromosomes and are transcribed with at least eight other genes (40,41), one of which encodes one of several variable transferrin receptors that confer different binding affinities for the transferrins of different mammals. Therefore, African trypanosomes combine antigenic variation of their surface coats with the ability to take up transferrin from their mammalian hosts (42).

A given VSG coat protein is encoded by a single *vsg* gene. Antigenic variation of VSG coats can occur by all the mechanisms described above, namely, transcriptional control, gene conversions, single crossover events between telomeric genes, and point mutations (37). A complete VSG gene conversion is usual in the early stages of infection, while partial replacement and point mutations that may generate further diversity are observed in the more chronic stages (43,44). Short blocks of sequence homology in the upstream and downstream regions of the donor and acceptor genes may be required for the recombination events, but the precise basis for these switching events remains unknown.

A possible mechanism may involve the unusual DNA base J, which is enriched in silent telomeric sites but is absent in expressed regions (45). Site-specific nucleases have not been described, but RAD51, an enzyme involved in DNA break repair and genetic exchange in other eukaryotes, may be involved (46). For transcriptional activation and silencing of *vsg* expression in the bloodstream forms of African trypanosomes, the presence of a certain sequence within the promoter may not be critical. When an expression site *vsg* promoter is replaced by ribosomal DNA promoter, *vsg* expression sites may still be silenced or activated (47).

### *Plasmodium falciparum*

During malaria infection, the apicomplexan parasites of the genus *Plasmodium* undergo repeated cycles of growth in erythrocytes. The species *P. falciparum* has strains that differ in several polymorphic proteins, but antigenic variation within a strain also occurs. The best-documented example of true antigenic variation is in the *P. falciparum*-infected erythrocyte membrane protein 1 (PfEMP1) antigens, which are expressed on the surface of the infected erythrocytes. Switching rates between PfEMP1 proteins may be as high as $10^{-2}$ per generation (Table) (48). By changing which PfEMP1 is expressed, the parasite evades the immune response directed against these immunodominant antigens. The PfEMP1 proteins also inhibit antigen presentation by dendritic cells and provide the means for the infected red cells to adhere to endothelium and extracellular matrix, thus avoiding clearance of the infected erythrocytes by the spleen (48-50).

The variable PfEMP1 proteins range from 200 to 350 kDa. Their extracellular region has variable adhesive domains that confer the parasite-infected erythrocytes with a particular binding specificity that can include the extracellular matrix protein thrombospondin and a variety of endothelial receptors such as CD36, vascular cell adhesion molecule-1 (VCAM-1), E-selectin (ELAM-1), and intercellular cell adhesion molecule type 1 (ICAM-1) (49,50). These adhesive phenotypes lead to the sequestration of infected erythrocytes in the brain, lungs, kidneys, liver, or other organs, thereby determining the clinical manifestations of malaria.

The PfEMP1 proteins are encoded by members of the *var* family of genes (49,51,52). Each parasite devotes approximately 2% to 6% of its genomic DNA to a repertoire of 50 to 150 *var* genes clustered near the ends of chromosomes. Transcription of the *var* genes can occur from expression sites internal on the chromosomes or near a chromosome telomere (53). Changes in *var* expression appear to occur in situ by recombination-independent mechanisms (51,52). Evidence indicates that a single *P. falciparum* simultaneously transcribes multiple *var* genes during its early ring stages, but in trophozoites, tighter transcriptional control results in the expression of a single PfEMP-1 on the surface of the host cell (54,55).

Two additional variant multigene families that, like PfEMP1, are expressed on the surface of infected red blood cells, induce specific antibodies, and undergo clonal variation have been described recently (56,57). These proteins are encoded by the *rif* and *STEVOR* genes, which are located near the telomeres that contain the *var* genes.

### *Babesia bovis*

Members of the genus *Babesia* cause one of the most common parasitic infections worldwide in wild and domestic animals. Some of the species, such as *B. microti,* have been transmitted to humans. Like *Plasmodium*, *Babesia* are intraerythrocytic parasites, but they are transmitted by ticks, not mosquitoes. While several multigene families have been described for various species of *Babesia*, clonal antigenic variation of *B. bovis*, a parasite of cattle, is best

Table. Vector-borne infections with antigenic variation

| Disease | Pathogen(s) | Vector | Variable antigens[a] |
|---|---|---|---|
| Relapsing fever | Several species of *Borrelia*, e.g., *B. hermsii* | Soft (argasid) ticks and body lice | Vlp & Vsp |
| Anaplasmosis | *Anaplasma marginale* | Hard (ixodid) ticks | MSP2 |
| African trypanosomiasis | African *Trypanosoma* spp., e.g., *T. brucei* | Tsetse fly | VSG |
| Malaria | *Plasmodium falciparum* | Mosquitoes | PfEMP1 |
| Babesiosis | *Babesia bovis* | Hard (ixodid) ticks | VESA1 |

[a]Vlp = variable large proteins; Vsp = variable small proteins; MSP2 = major surface protein 2; VSG = variant-specific glycoprotein; MfEMP1 = *P. falciparum* erythrocyte membrane protein 1; VESA1 = variant erythrocyte surface antigen 1.

documented (58). The variant erythrocyte surface antigen (VESA1) of *B. bovis* is a heterodimeric protein expressed on the surface of infected red blood cells. The rapid variation of these polymorphic proteins likely contributes to chronic infection in cattle by prolonging the parasite's survival through immune evasion and sequestration of the infected red blood cells in peripheral organs (58). The VESA1 proteins, which have an approximate molecular weight of 128 kDa (59), are expressed on the external tips of the membrane knobs of infected erythrocytes. Their cytoadhesive phenotype depends on the antigenic and structural changes of the VESA1 proteins (60). The gene encoding the VESA1a subunit has been recently shown to belong to the *ves* multigene family (61). The predicted protein does not seem to have cleavable signal sequence, but it does have a predicted transmembrane segment and a cysteine/lysine-rich domain (61). The molecular events that determine the switching mechanism in *B. babesia* are unknown.

### Conclusions

The requirement for vector transmission of these infectious pathogens provides a powerful selection for mechanisms that prolong parasitemia. Through convergent evolution, several vector-borne pathogens have arrived at the same strategy of antigenic variation to achieve this goal. The similarity in the genetic mechanisms that such unrelated pathogens as African trypanosomes and relapsing fever *Borrelia* spp. use for antigenic variation is remarkable.

Antigenic variation has important implications for the development of vaccines against these pathogens. If the variable antigen is to be the target of immunoprophylaxis, the vaccine would likely need to be multivalent, perhaps to the point of impracticality. If the infected host animal has not solved the problem of identifying an antigen that is conserved among the variants, thereby neutralizing the infection earlier, how can vaccine developers hope to do this?

A possible way to meet this challenge is to focus on the function domains of the variable proteins. The variable antigens of both the bacterial and parasite pathogens have other roles in pathogenesis besides immune evasion. These include tissue tropism, shielding of adjacent molecules, inhibition of phagocytosis, modulation of antigen presentation, and selective adherence. Certain regions of the variable protein may be irrelevant for these functions of the pathogen, and consequently the encoding DNA sequences could be highly divergent among alleles. On the other hand, the regions conferring these functions would likely be more constrained in structure and thus comparatively more susceptible to cross-reacting antibodies.

Another possible way to meet the challenge of antigenic variation is to focus on the vector-specific surface antigens of these pathogens. The repertoire expressed in the arthropod vector, which lacks an adaptive immune system, is generally more limited than that expressed in the vertebrate host. The Lyme disease vaccine is an example of successful targeting of a vector-specific protein. Although *B. burgdorferi* has not yet been proven to undergo true antigenic variation, there is considerable diversity in the *ospC* sequences that define strain identity within a given area in which transmission to humans occurs. A vaccine based on OspC would likely need to be multivalent. In contrast, *B. burgdorferi*'s OspA protein (62), the sole protein in the vaccine, is natively expressed in the tick's midgut but usually not during infection of mammals (63). Perhaps because of OspA's infrequent encounters with the mammalian adaptive immune system in nature, there is little divergence in *ospA* sequences between strains of *B. burgdorferi* (64). The OspA-based vaccine apparently works by eliciting antibodies that kill or inhibit the spirochetes in the tick, before expression of the more polymorphic *ospC* and *vlsE* genes in the mammalian host (65).

This research was supported by grants AI24424 and AI37248 from the National Institutes of Health and a grant from the Centers for Disease Control and Prevention (A.G.B.) and by grants from the Instituto Colombiano para el Desarrollo de la Ciencia y la Tecnología Francisco José de Caldas, Fogarty International, and the International Society for Infectious Diseases (B.I.R.).

Dr. Barbour is professor of medicine and microbiology & molecular genetics at the University of California Irvine. His research focuses on the molecular pathogenesis of relapsing fever and Lyme disease and the prevention of tick-borne diseases.

Dr. Restrepo is head of the molecular parasitology group at the Corporación para Investigaciones Biológicas in Medellín, Colombia. Her current research interests focus on the molecular and immunologic aspects of neurocysticercosis.

### References

1. Wang IN, Dykhuizen DE, Qiu W, Dunn JJ, Bosler EM, Luft BJ. Genetic diversity of ospC in a local population of *Borrelia burgdorferi* sensu stricto. Genetics 1999;151:15-30.
2. Stevenson B, Bockenstedt LK, Barthold SW. Expression and gene sequence of outer surface protein C of *Borrelia burgdorferi* reisolated from chronically infected mice. Infect Immun 1994;62:3568-71.
3. Beale GH, J.F. W. Antigenic variation in unicellular organisms. Ann Rev Microbiol 1961;15:263-96.
4. Barbour AG, Hayes SF. Biology of Borrelia species. Microbiol Rev 1986;50:381-400.
5. Zuckert WR, Meyer J, Barbour AG. Comparative analysis and immunological characterization of the Borrelia Bdr protein family. Infect Immun 1999;67:3257-66.
6. de la Vega I, Gonzalez A, Blasco R, Calvo V, Vinuela E. Nucleotide sequence and variability of the inverted terminal repetitions of African swine fever virus DNA. Virology 1994;201:152-6.
7. Turner CM. Antigenic variation in *Trypanosoma brucei* infections: an holistic view. J Cell Sci 1999;112:3187-92.
8. Barbour AG. Immunobiology of relapsing fever. Contrib Microbiol Immunol 1987;8:125-37.
9. Vickerman K. Trypanosome sociology and antigenic variation. Parasitology 1989;99:S37-S47.
10. Deitsch KW, Moxon ER, Wellems TE. Shared themes of antigenic variation and virulence in bacterial, protozoal, and fungal infections. Microbiol Mol Biol Rev 1997;61:281-93.
11. Hinnebusch BJ, Barbour AG, Restrepo BI, Schwan TG. Population structure of the relapsing fever spirochete *Borrelia hermsii* as indicated by polymorphism of two multigene families that encode immunogenic outer surface lipoproteins. Infect Immun 1998;66:432-40.
12. Barbour AG. Linear DNA of Borrelia species and antigenic variation. Trends Microbiol 1993;1:236-9.
13. Stoenner HG, Dodd T, Larsen C. Antigenic variation of *Borrelia hermsii*. J Exp Med 1982;156:1297-311.
14. Barbour AG, Tessier SL, Stoenner HG. Variable major proteins of *Borrellia hermsii*. J Exp Med 1982;156:1312-24.
15. Burman N, Bergstrom S, Restrepo BI, Barbour AG. The variable antigens Vmp7 and Vmp21 of the relapsing fever bacterium *Borrelia hermsii* are structurally analogous to the VSG proteins of the African trypanosome. Mol Microbiol 1990;4:1715-26.
16. Restrepo BI, Kitten T, Carter CJ, Infante D, Barbour AG. Subtelomeric expression regions of *Borrelia hermsii* linear plasmids are highly polymorphic. Mol Microbiol 1992;6:3299-311.
17. Vidal V, Scragg IG, Cutler SJ, Rockett KA, Fekade D, Warrell DA, et al. Variable major lipoprotein is a principal TNF-inducing factor of louse-borne relapsing fever. Nat Med 1998;4:1416-20.
18. Shamaei-Tousi A, Martin P, Bergh A, Burman N, Brännström T, Bergström S. Erythrocyte-aggregating relapsing fever spirochete *Borrelia crocidurae* induces formation of microemboli. J Infect Dis 1999;180:1929-38.
19. Pennington PM, Cadavid D, Bunikis J, Norris SJ, Barbour AG. Extensive interplasmidic duplications change the virulence phenotype of the relapsing fever agent *Borrelia turicatae*. Mol Microbiol 1999;34:1120-32.
20. Cadavid D, Thomas DD, Crawley R, Barbour AG. Variability of a bacterial surface protein and disease expression in a possible mouse model of systemic Lyme borreliosis. J Exp Med 1994;179:631-42.
21. Pennington PM, Allred CD, West CS, Alvarez R, Barbour AG. Arthritis severity and spirochete burden are determined by serotype in the *Borrelia turicatae*-mouse model of Lyme disease. Infect Immun 1997;65:285-92.
22. Kitten T, Barbour AG. Juxtaposition of expressed variable antigen genes with a conserved telomere in the bacterium *Borrelia hermsii*. Proc Natl Acad Sci U S A 1990;87:6077-81.
23. Barbour AG, Burman N, Carter CJ, Kitten T, Bergstrom S. Variable antigen genes of the relapsing fever agent *Borrelia hermsii* are activated by promoter addition. Mol Microbiol 1991;5:489-93.
24. Kitten T, Barrera AV, Barbour AG. Intragenic recombination and a chimeric outer membrane protein in the relapsing fever agent *Borrelia hermsii*. J Bacteriol 1993;175:2516-22.
25. Restrepo BI, Carter CJ, Barbour AG. Activation of a vmp pseudogene in *Borrelia hermsii*: an alternate mechanism of antigenic variation during relapsing fever. Mol Microbiol 1994;13:287-99.
26. Restrepo BI, Barbour AG. Antigen diversity in the bacterium *B. hermsii* through "somatic" mutations in rearranged vmp genes. Cell 1994;78:867-76.
27. Carter CJ, Bergstrom S, Norris SJ, Barbour AG. A family of surface-exposed proteins of 20 kilodaltons in the genus Borrelia. Infect Immun 1994;62:2792-9.
28. Barbour AG, Carter CJ, Sohaskey CD. Surface protein variation by expression site switching in the relapsing fever agent *Borrelia hermsii*. Infect Immun. In press 2000.
29. Schwan TG, Hinnebusch BJ. Bloodstream- versus tick-associated variants of a relapsing fever bacterium. Science 1998;280:1938-40.
30. Zhang JR, Hardham JM, Barbour AG, Norris SJ. Antigenic variation in Lyme disease borreliae by promiscuous recombination of VMP-like sequence cassettes. Cell 1997;89:275-85.
31. Zhang JR, Norris SJ. Kinetics and in vivo induction of genetic variation of vlsE in *Borrelia burgdorferi*. Infect Immun 1998;66:3689-97.
32. Kieser ST, Eriks IS, Palmer GH. Cyclic rickettsemia during persistent *Anaplasma marginale* infection of cattle. Infect Immun 1990;58:1117-9.
33. Palmer GH, Abbott JR, French DM, McElwain TF. Persistence of *Anaplasma ovis* infection and conservation of the msp-2 and msp-3 multigene families within the genus *Anaplasma*. Infect Immun 1998;66:6035-9.
34. Sulsona CR, Mahan SM, Barbet AF. The map1 gene of *Cowdria ruminantium* is a member of a multigene family containing both conserved and variable genes. Biochem Biophys Res Commun 1999;257:300-5.
35. Zhi N, Ohashi N, Rikihisa Y. Multiple p44 genes encoding major outer membrane proteins are expressed in the human granulocytic ehrlichiosis agent. J Biol Chem 1999;274:17828-36.
36. French DM, Brown WC, Palmer GH. Emergence of *Anaplasma marginale* antigenic variants during persistent rickettsemia. Infect Immun 1999;67:5834-40.

37. Rudenko G, Cross M, Borst P. Changing the end: antigenic variation orchestrated at the telomeres of African trypanosomes. Trends Microbiol 1998;6:113-6.
38. Lamont GS, Tucker RS, Cross GA. Analysis of antigen switching rates in *Trypanosoma brucei*. Parasitology 1986;92:355-67.
39. Turner CM. The rate of antigenic variation in fly-transmitted and syringe-passaged infections of *Trypanosoma brucei*. FEMS Microbiol Lett 1997;153:227-31.
40. Pays E, Tebabi P, Pays A, Coquelet H, Revelard P, Salmon D, et al. The genes and transcripts of an antigen gene expression site from *T. brucei*. Cell 1989;57:835-45.
41. Lips S, Revelard P, Pays E. Identification of a new expression site-associated gene in the complete 30.5 kb sequence from the AnTat 1.3A variant surface protein gene expression site of *Trypanosoma brucei*. Mol Biochem Parasitol 1993;62:135-7.
42. Bitter W, Gerrits H, Kieft R, Borst P. The role of transferrin-receptor variation in the host range of *Trypanosoma brucei*. Nature 1998;391:499-502.
43. Baltz T, Giroud C, Bringaud F, Eisen H, Jacquemot C, Roth CW. Exposed epitopes on a *Trypanosoma equiperdum* variant surface glycoprotein altered by point mutations. EMBO J 1991;10:1653-9.
44. Lu Y, Alarcon CM, Hall T, Reddy LV, Donelson JE. A strand bias occurs in point mutations associated with variant surface glycoprotein gene conversion in *Trypanosoma rhodesiense*. Mol Cell Biol 1994;14:3971-80.
45. Gommers-Ampt J, Lutgerink J, Borst P. A novel DNA nucleotide in *Trypanosoma brucei* only present in the mammalian phase of the life-cycle. Nucleic Acids Res 1991;19:1745-51.
46. McCulloch R, Barry JD. A role for RAD51 and homologous recombination in *Trypanosoma brucei* antigenic variation. Genes Dev 1999;13:2875-88.
47. Rudenko G, Blundell PA, Dirks-Mulder A, Kieft R, Borst P. A ribosomal DNA promoter replacing the promoter of a telomeric VSG gene expression site can be efficiently switched on and off in *T. brucei*. Cell 1995;83:547-53.
48. Roberts DJ, Craig AG, Berendt AR, Pinches R, Nash G, Marsh K, et al. Rapid switching to multiple antigenic and adhesive phenotypes in malaria [see comments]. Nature 1992;357:689-92.
49. Baruch DI, Pasloske BL, Singh HB, Bi X, Ma XC, Feldman M, et al. Cloning the *P. falciparum* gene encoding PfEMP1, a malarial variant antigen and adherence receptor on the surface of parasitized human erythrocytes. Cell 1995;82:77-87.
50. Gardner JP, Pinches RA, Roberts DJ, Newbold CI. Variant antigens and endothelial receptor adhesion in *Plasmodium falciparum*. Proc Natl Acad Sci U S A 1996;93:3503-8.
51. Smith JD, Chitnis CE, Craig AG, Roberts DJ, Hudson-Taylor DE, Peterson DS, et al. Switches in expression of *Plasmodium falciparum* var genes correlate with changes in antigenic and cytoadherent phenotypes of infected erythrocytes. Cell 1995;82:101-10.
52. Su XZ, Heatwole VM, Wertheimer SP, Guinet F, Herrfeldt JA, Peterson DS, et al. The large diverse gene family var encodes proteins involved in cytoadherence and antigenic variation of *Plasmodium falciparum*-infected erythrocytes. Cell 1995;82:89-100.
53. Hernandez-Rivas R, Mattei D, Sterkers Y, Peterson DS, Wellems TE, Scherf A. Expressed var genes are found in *Plasmodium falciparum* subtelomeric regions. Mol Cell Biol 1997;17:604-11.
54. Chen Q, Fernandez V, Sundstrom A, Schlichtherle M, Datta S, Hagblom P, et al. Developmental selection of var gene expression in *Plasmodium falciparum*. Nature 1998;394:392-5.
55. Scherf A, Hernandez-Rivas R, Buffet P, Bottius E, Benatar C, Pouvelle B, et al. Antigenic variation in malaria: in situ switching, relaxed and mutually exclusive transcription of var genes during intra-erythrocytic development in *Plasmodium falciparum*. EMBO J 1998;17:5418-26.
56. Kyes SA, Rowe JA, Kriek N, Newbold CI. Rifins: a second family of clonally variant proteins expressed on the surface of red cells infected with *Plasmodium falciparum*. Proc Natl Acad Sci U S A 1999;96:9333-8.
57. Fernandez V, Hommel M, Chen Q, Hagblom P, Wahlgren M. Small, clonally variant antigens expressed on the surface of the *Plasmodium falciparum*-infected erythrocyte are encoded by the rif gene family and are the target of human immune responses. J Exp Med 1999;190:1393-404.
58. Allred DR, Cinque RM, Lane TJ, Ahrens KP. Antigenic variation of parasite-derived antigens on the surface of *Babesia bovis*-infected erythrocytes. Infect Immun 1994;62:91-8.
59. O'Connor RM, Lane TJ, Stroup SE, Allred DR. Characterization of a variant erythrocyte surface antigen (VESA1) expressed by *Babesia bovis* during antigenic variation. Mol Biochem Parasitol 1997;89:259-70.
60. O'Connor RM, Allred DR. Selection of *Babesia bovis*-infected erythrocytes for adhesion to endothelial cells coselects for altered variant erythrocyte surface antigen isoforms. J Immunol 2000;164:2037-45.
61. Allred DR, Carlton JM, Satcher RL, Long JA, Brown WC, Patterson PE, et al. The ves multigene family of *B. bovis* encodes components of rapid antigenic variation at the infected erythrocyte surface. Mol Cell 2000;5:153-62.
62. Barbour AG, Tessier SL, Todd WJ. Lyme disease spirochetes and ixodid tick spirochetes share a common surface antigenic determinant defined by a monoclonal antibody. Infect Immun 1983;41:795-804.
63. Schwan TG, Piesman J. Temporal changes in outer surface proteins A and C of the Lyme disease-associated spirochete, *Borrelia burgdorferi*, during the chain of infection in ticks and mice. J Clin Microbiol 2000;38:382-8.
64. Jonsson M, Noppa L, Barbour AG, Bergstrom S. Heterogeneity of outer membrane proteins in *Borrelia burgdorferi*: comparison of osp operons of three isolates of different geographic origins. Infect Immun 1992;60:1845-53.
65. de Silva AM, Zeidner NS, Zhang Y, Dolan MC, Piesman J, Fikrig E. Influence of outer surface protein A antibody on Borrelia burgdorferi within feeding ticks. Infect Immun 1999;67:30-5.
